# Prognostic utility and characterization of left ventricular hypertrophy using global thickness

**DOI:** 10.1038/s41598-023-48173-7

**Published:** 2023-12-20

**Authors:** Magnus Lundin, Einar Heiberg, David Nordlund, Tom Gyllenhammar, Katarina Steding-Ehrenborg, Henrik Engblom, Marcus Carlsson, Dan Atar, Jesper van der Pals, David Erlinge, Rasmus Borgquist, Ardavan Khoshnood, Ulf Ekelund, Jannike Nickander, Raquel Themudo, Sabrina Nordin, Rebecca Kozor, Anish N. Bhuva, James C. Moon, Eva Maret, Kenneth Caidahl, Andreas Sigfridsson, Peder Sörensson, Erik B. Schelbert, Håkan Arheden, Martin Ugander

**Affiliations:** 1 Department of Clinical Physiology, Karolinska University Hospital, and Karolinska Institutet, Stockholm, Sweden; 2 Department of Clinical Sciences Lund, Clinical Physiology, Lund University, Skåne University Hospital, Lund, Sweden; 3 Wallenberg Center for Molecular Medicine, Lund University, Lund, Sweden; 4 Department of Health Sciences, Physiotherapy, Lund University, Lund, Sweden; 5 Department of Cardiology, and Institute of Clinical Medicine, Oslo University Hospital Ulleval, University of Oslo, Oslo, Norway; 6 Arrhythmia Clinic, Skåne University Hospital, and Department of Cardiology, Clinical Sciences, Lund University, Lund, Sweden; 7 Department of Clinical Sciences, Cardiology, Lund University, Skåne University Hospital, Lund, Sweden; 8 Department of Clinical Sciences, Emergency and Internal Medicine, Lund University, Skåne University Hospital, Lund, Sweden; 9 Institute of Cardiovascular Science, University College London, London, UK; 10 Kolling Institute, Royal North Shore Hospital, and University of Sydney, Sydney, Australia; 11 Department of Cardiology, Barts Heart Centre, London, UK; 12 Institute of Medicine, University of Gothenburg and Clinical Physiology, Sahlgrenska University Hospital, Gothenburg, Sweden; 13 Department of Cardiology, Karolinska University Hospital, Stockholm, Sweden; 14 Department of Molecular Medicine and Surgery, Karolinska Institutet, Stockholm, Sweden; 15 University of Pittsburgh Medical Center, Pittsburgh, PA USA; 16 Royal North Shore Hospital, University of Sydney, Kolling Building, Level 12, Room 612017, St Leonards, NSW 2065 Australia

**Keywords:** Medical imaging, Cardiology

## Abstract

Cardiovascular magnetic resonance (CMR) can accurately measure left ventricular (LV) mass, and several measures related to LV wall thickness exist. We hypothesized that prognosis can be used to select an optimal measure of wall thickness for characterizing LV hypertrophy. Subjects having undergone CMR were studied (cardiac patients, n = 2543; healthy volunteers, n = 100). A new measure, global wall thickness (GT, GTI if indexed to body surface area) was accurately calculated from LV mass and end-diastolic volume. Among patients with follow-up (n = 1575, median follow-up 5.4 years), the most predictive measure of death or hospitalization for heart failure was LV mass index (LVMI) (hazard ratio (HR)[95% confidence interval] 1.16[1.12–1.20], p < 0.001), followed by GTI (HR 1.14[1.09–1.19], p < 0.001). Among patients with normal findings (n = 326, median follow-up 5.8 years), the most predictive measure was GT (HR 1.62[1.35–1.94], p < 0.001). GT and LVMI could characterize patients as having a normal LV mass and wall thickness, concentric remodeling, concentric hypertrophy, or eccentric hypertrophy, and the three abnormal groups had worse prognosis than the normal group (p < 0.05 for all). LV mass is highly prognostic when mass is elevated, but GT is easily and accurately calculated, and adds value and discrimination amongst those with normal LV mass (early disease).

## Introduction

Accurate characterization of left ventricular (LV) hypertrophy (LVH) is important since an increased LV mass (LVM) due to various forms of hypertrophy and remodeling is both a risk factor for cardiovascular disease and also modifiable^1–3^. Echocardiographic measurements of end-diastolic wall thickness and cavity size lead to the standardized terminology of normal wall thickness and size, concentric hypertrophy, eccentric hypertrophy, and concentric remodeling^4^. Cardiovascular magnetic resonance (CMR) provides additional accuracy^5^ and precision^6^, via direct measurement of LV myocardial volumes and mass.

However, LVM alone does not allow for an assessment of wall thickness. There are several measures calculated from LVM and LV end-diastolic volume (LVEDV) that are related to wall thickness, such as mass:volume ratio (MVR = LVM/LVEDV) and concentricity^0.67^ (= LVM/LVEDV^2/3^). We hypothesized that a wall thickness-related measure could be used in combination with LVMI to characterize patients as having concentric remodeling (normal LVMI but high wall thickness), or as having hypertrophy (increased LVMI) that is either concentric (high wall thickness) or eccentric (low wall thickness). We further hypothesized that a measure such as global wall thickness (GT), which estimates the average wall thickness of the whole left ventricle in mm, could be easily and accurately calculated from LVM and LVEDV without the need for any special software. Finally, we also hypothesized that GT would have prognostic utility. This study sought to compare GT to other measures of LV hypertrophy by using prognostic association to evaluate which wall thickness-related measure would have greatest clinical relevance.

Therefore, the aims of the study were: (a) to use CMR to describe a new measure of global wall thickness (GT) that could be easily calculated, (b) to determine relative prognostic performance for LV mass, GT, and several other wall thickness-related measures, and (c) to classify LVH based on the measures with the highest prognostic associations.

## Results

In the derivation subset (*n* = 269) of the derivation/validation cohort, the optimized equation for the calculated global wall thickness (GT) was found to be:

1 $$GT = 0.0{5} + {1}.{6}0 \cdot LVM^{{0.{84}}} \cdot LVEDV^{{ - 0.{49}}}$$

where GT is the global wall thickness in mm, LVM is left ventricular mass in grams, and LVEDV is left ventricular end-diastolic volume in milliliters. This equation can be used for any CMR results where only the values for LVM and LVEDV are available. Consequently, no special plugin or software is needed to calculate GT in clinical routine.

**Figure 1:**
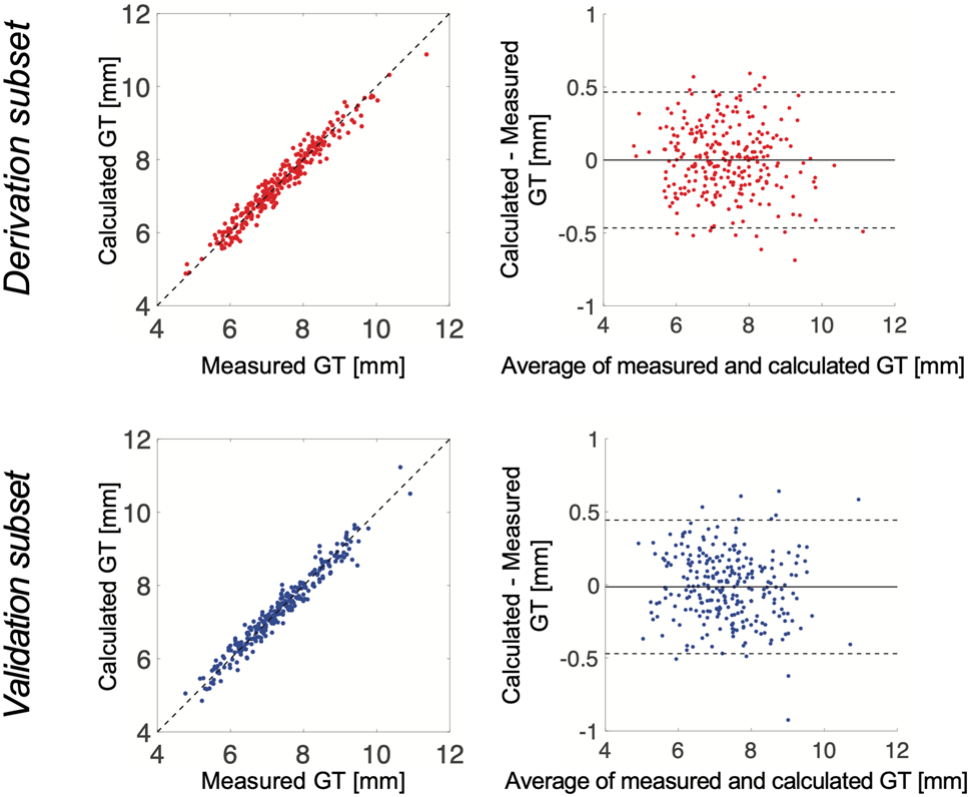
Plots of the calculated vs. measured global wall thickness (GT) in mm. GT was measured using the method illustrated in Fig. 7, and was estimated using the derived Eq. (1). Top left: Correlation plot for the derivation subset (*n* = 269), *R*^2^ = 0.95, bias 0.00 ± 0.24 mm, identity line shown dashed. Top right: Bland–Altman plot for the derivation subset of the cohort. Solid line shows mean difference and dashed lines show ± 1.96 standard deviations. Bottom left: Correlation plot for the separate validation subset (*n* = 268), *R*^2^ = 0.95, *p* < 0.001, bias 0.01 ± 0.23 mm, identity line shown dashed. Bottom right: Bland–Altman plot for the validation subset of the cohort. Solid line shows mean difference and dashed lines show ± 1.96 standard deviations. *GT* global wall thickness.

For the derivation subset, the model had an expectedly high correlation (*R*^2^ = 0.95, *p* < 0.001) with no bias (0.00 ± 0.24 mm), see Fig. 1, upper panels. When applied to the separate *validation subset* of the cohort (*n* = 268) model performance was preserved (*R*^2^ = 0.95, *p* < 0.001, bias 0.01 ± 0.23 mm), see Fig. 1, lower panels. The wide range of values for LV mass and volumes in the various patient groups of the derivation and validation cohorts (see Tables 1, 2) imply that the derived equation should be valid for a wide range of combinations of LV size, mass and wall thickness.

**Table 1:** Characteristics for the female subjects of the *derivation/validation cohort* (n = 157). Data shown as median and interquartile range where * denotes *p* < 0.05 compared to the healthy volunteers.

*Females*	Healthy volunteers		Athletes		CRT		Infarction		Cardiac syndrome X	
Number, *n*	27		41		7		64		18	
Age, years	36	(26–60)	22	(19–26)*	66	(63–74)*	69.5	(60–75)*	69.5	(57–74)*
Length, cm	169	(165–170)	170	(167–174)	163	(159–174)	165	(160–171)	163	(160–168)*
Weight, kg	66	(59–70)	64	(60–70)	67	(59–80)	73	(66–80)*	71	(64–79)
BSA, m^2^	1.8	(1.7–1.8)	1.7	(1.7–1.8)	1.8	(1.6–1.9)	1.8	(1.7–1.9)*	1.8	(1.7–1.9)
BMI, kg/m^2^	23	(21–25)	22	(21–23)	25	(22–30)	26	(24–28)*	26	(25–28)*
LVEF, %	61	(59–63)	57	(56–59)*	26	(18–36)*	52	(45–58)*	68	(66–70)*
LVEDV, ml	156	(131–169)	188	(175–204)*	300	(196–392)*	131	(114–170)*	126	(118–144)*
LVESV, ml	61	(50–72)	82	(73–88)*	215	(124–321)*	65	(49–81)	39	(35–47)*
LVSV, ml	91	(83–104)	107	(99–116)*	72	(56–106)	69	(57–83)*	87	(78–98)
LVM, g	88	(74–94)	111	(105–117)*	167	(107–210)*	98	(86–113)*	90	(81–98)
LVEDVI, ml/m^2^	90	(77–97)	109	(99–115)*	181	(117–236)*	75	(64–88)*	70	(66–83)*
LVESVI, ml/m^2^	28	(36–39)	41	(47–49)*	67	(120–193)*	28	(36–45)	20	(22–27)*
LVSVI, ml/m^2^	53	(48–59)	62	(58–65)*	43	(32–58)	40	(32–46)*	47	(45–54)
LVMI, ml/m^2^	51	(42–54)	64	(57–69)*	93	(62–115)*	55	(48–62)*	51	(46–55)

*BMI* body mass index, *BSA* body surface area, *CRT* cardiac resynchronization therapy-candidates (patients with heart failure), *LVEDV(I)* left ventricular end-diastolic volume (indexed to BSA), *LVEF* left ventricular ejection fraction, *LVESV(I)* end-systolic volume (indexed to BSA), *LVM(I)* left ventricular mass (indexed to BSA), *LVSV(I)* left ventricular stroke volume (indexed to BSA).

**Table 2:** Characteristics for the male subjects of the *derivation/validation cohort* (*n* = 380). Data shown as median and interquartile range where * denotes *p* < 0.05 compared to the healthy volunteers.

*Males*	Healthy volunteers		Athletes		CRT		Infarction		Cardiac syndrome X	
Number, *n*	50		45		28		236		21	
Age, years	34	(27–51)	26	(21–33)*	69	(65–74)*	59	(51–68)*	65	(60–70)*
Length, cm	181	(178–183)	186	(181–188)*	176	(171–182)*	177	(172–180)*	177	(174–182)*
Weight, kg	80.5	(74–88)	82	(78–87)	82.5	(76–92)	84	(77–92)	85	(80–95)
BSA, m^2^	2.0	(1.9–2.1)	2.0	(2.0–2.1)	2.0	(1.9–2.2)	2.0	(1.9–2.1)	2.1	(1.9–2.2)
BMI, kg/m^2^	24	(23–27)	24	(23–25)	27	(24–30)*	27	(25–29)*	27	(25–29)*
LVEF, %	59	(55–62)	55	(52–59)*	26	(20–34)*	48	(42–55)*	63	(59–68)*
LVEDV, ml	194	(172–215)	252	(225–273)*	331	(273–369)*	181	(157–204)*	171	(162–220)
LVESV, ml	78	(72–89)	112	(97–127)*	243	(177–281)*	93	(76–115)*	62	(56–92)*
LVSV, ml	114	(104–128)	138	(130–150)*	81	(65–96)*	85	(74–98)*	108	(89–124)
LVM, g	123	(112–136)	160	(144–179)*	185	(150–215)*	133	(117–147)*	138	(120–161)*
LVEDVI, ml/m^2^	96	(89–106)	123	(117–132)*	156	(136–188)*	90	(80–102)*	84	(76–104)*
LVESVI, ml/m^2^	40	(35–45)	55	(48–61)*	114	(89–147)*	46	(38–57)*	29	(25–43)*
LVSVI, ml/m^2^	57	(51–64)	69	(65–72)*	42	(33–50)*	43	(37–49)*	51	(48–59)*
LVMI, ml/m^2^	62	(57–69)	79	(72–87)*	90	(75–102)*	67	(58–73)*	68	(61–77)

*BMI* body mass index, *BSA* body surface area, *CRT* cardiac resynchronization therapy-candidates (patients with heart failure), *LVEDV(I)* left ventricular end-diastolic volume (indexed to BSA), *LVEF* left ventricular ejection fraction, *LVESV(I)* end-systolic volume (indexed to BSA), *LVM(I)* left ventricular mass (indexed to BSA), *LVSV(I)* left ventricular stroke volume (indexed to BSA).

### Prognostic analysis

In the *survival cohort* (*n* = 1575, 42% female, follow-up 5.4 [3.9–6.4] years), univariable Cox regression showed that, apart from age at CMR and presence of hypertension, the parameter with the highest prognostic value for hospitalization for heart failure (HHF) or death was LVMI (χ^2^ 66.7, *p* < 0.001) followed by GTI (χ^2^ 37.3, *p* < 0.001) and GT (χ^2^ 33.1, *p* < 0.001), see Table 3. In multivariable analysis including LVMI and GTI, LVMI was associated with outcomes (*p* < 0.001) and GTI was not (*p* = 0.60).

**Table 3:** Prognostic results, all patients.

Patients (events)	1575 (351)			
Follow-up, years	5.4 (3.9–6.4)			
	*χ*^2^	Univariable analysis		*p*
		HR [95% CI]		
Age at CMR, years	97.9	1.04	[1.03–1.05]	< 0.001
LVMI, g/m^2^	66.7	1.16	[1.12–1.20]	< 0.001
Hypertension	52.8	2.3	[1.8–2.9]	< 0.001
GTI, mm/m^2^	37.3	1.14	[1.09–1.19]	< 0.001
GT, mm	33.1	1.12	[1.08–1.17]	< 0.001
Conc067, g/ml^2/3^	26.7	1.10	[1.06–1.15]	< 0.001
MVR, g/ml	7.5	1.05	[1.01–1.09]	0.006
RI, ml^1/3^/mm	3.7	-		0.053

Results from the Cox regression for risk of death or hospitalization for heart failure for all patients of the *survival cohort.* Data are presented as *χ*^2^ value, hazard ratio (HR) per standard deviation increase, and *p* value, ordered in decreasing *χ*^2^ values; follow-up time is presented as median (IQR).

*CI* confidence interval, *Conc067* left ventricular concentricity^0.67^ (left ventricular mass/end-diastolic volume^0.67^), *GT* global wall thickness, *GTI* global wall thickness index, *HR* hazard ratio, *IQR* inter-quartile range, *LVEDV* left ventricular end-diastolic volume, *LVMI* left ventricular mass index, *MVR* mass:volume ratio, *RI* remodeling index ([LVEDV]^(1/3)^/max wall thickness).

In the subgroup with normal findings (no LGE, and findings within the normal range per sex for LVEDVI, LVMI, and LVEF, *n* = 326, 45% female, follow-up 5.8 [5.0–6.7] years), the parameters with the highest prognostic value for HHF or death were GT (χ^2^ 26.8, *p* < 0.001), and concentricity^0.67^ (χ^2^ 26.6, *p* < 0.001), and these were more prognostic than both hypertension (χ^2^ 23.5, *p* < 0.001) and age at CMR (χ^2^ 14.5, *p* < 0.001), see Table 4. In multivariable analysis including LVMI and GT, LVMI was not associated with outcomes (*p* = 0.70) but GT was (*p* = 0.01).

**Table 4:** Prognostic results, patients with normal findings.

Patients (events)	326 (29)			
Follow-up, years	5.8 (5.0–6.7)			
	*χ*^2^	Univariable analysis		*p*
		HR [95% CI]		
GT, mm	26.8	1.62	[1.35–1.94]	< 0.001
Conc067, g/ml^2/3^	26.6	1.58	[1.33–1.88]	< 0.001
Hypertension	23.5	9.2	[3.8–22.7]	< 0.001
MVR, g/ml	21.9	1.57	[1.30–1.90]	< 0.001
RI, ml^1/3^/mm	18.7	0.54	[0.41–0.72]	< 0.001
LVMI, g/m^2^	16.0	2.05	[1.44–2.92]	< 0.001
Age at CMR, years	14.5	1.05	[1.02–1.08]	< 0.001
GTI, mm/m^2^	7.2	1.46	[1.11–1.92]	0.007

Results from the Cox regression for risk of death or hospitalization for heart failure for patients in the survival cohort with normal findings (LVMI, LVEDVI and LVEF within normal range per sex and normal LGE findings). Data are presented as *χ*^2^ value, hazard ratio (HR) per standard deviation increase, and *p* value, ordered in decreasing *χ*^2^ values; follow-up time is presented as median (IQR). *CI* confidence interval, *Conc067* left ventricular concentricity^0.67^ (left ventricular mass/end-diastolic volume^0.67^), *GT* global wall thickness, *GTI* global wall thickness index, *HR* hazard ratio, *IQR* inter-quartile range, *LVEDV* left ventricular end-diastolic volume, *LVEF* left ventricular ejection fraction, *LVMI* left ventricular mass index, *MVR* mass:volume ratio, *RI* remodeling index ([LVEDV](1/3)/max wall thickness).

### Test–retest

In the test–retest cohort (*n* = 101), the test–retest variability was lowest for GT (4.2%) and the highest variability was found for LVEDV and mass:volume ratio (6.1% and 6.2%, respectively, *p* < 0.001 for both versus GT).

### Normal ranges

In the mixed cohort, normal calculated GT (based on healthy volunteers (*n* = 99, 35% female), was 5.9 ± 0.6 mm for females and 7.2 ± 0.7 mm for males. This corresponds to a GT normal range of 4.8–7.1 mm for females and 5.8–8.5 mm for males. All patient groups, as well as the athletes, had a higher GT than healthy volunteers for both sexes (*p* < 0.02 for all groups separately). Patient characteristics of the mixed cohort are presented in Tables 5 and 6.

**Table 5:** Characteristics and results for the female subjects of the *mixed cohort*.

*Females*	Healthy volunteers		Fabry		LVH	
Number, *n*	35		86		60	
Age, years	29	(23–49)	45	(33–55)*	61	(47–68)*
Height, cm	169	(164–171)	164	(158–170)*	166	(162–170)
Weight, kg	65	(60–70)	66	(61–76)	68	(61–78)
BSA, m^2^	1.8	(1.7–1.8)	1.7	(1.6–1.9)	1.7	(1.7–1.9)
BMI, kg/m^2^	23	(21–25)	24	(22–28)*	25	(21–28)
LVEDV, ml	152	(131–169)	120	(107–134)*	182	(138–232)*
LVEDVI, ml/m^2^	88	(77–95)	68	(61–76)*	100	(80–127)*
LVESV, ml	60	(52–70)	29	(22–35)*	89	(63–147)*
LVESVI, ml/m^2^	36	(29–39)	17	(13–21)*	49	(35–78)*
LVSV, ml	89	(82–97)	91	(81–101)	82	(64–103)*
LVSVI, ml/m^2^	51	(47–57)	52	(47–56)	46	(37–53)*
LVEF, %	60	(58–63)	76	(71–80)*	46	(35–61)*
LVM, g	89	(77–97)	104	(85–124)*	149	(134–175)*
LVMI, g/m^2^	51	(45–56)	56	(49–71)*	83	(77–96)*
GT, mm	6.0	(5.6–6.3)	7.5	(6.4–9.0)*	8.7	(7.9–9.5)*
GTI, mm/m^2^	3.4	(3.1–3.6)	4.2	(3.7–5.2)*	4.8	(4.3–5.5)*

Only the groups that were changed from the derivation/validation cohort are shown. Characteristics for the healthy volunteers and the patient groups shown as median and interquartile range, and * denotes *p* < 0.05 compared to the healthy volunteers. *BMI* body mass index, *BSA* body surface area, *GT(I)* global wall thickness (indexed to BSA), *LVEDV(I*) left ventricular end-diastolic volume (indexed to BSA), *LVEF* left ventricular ejection fraction, *LVESV(I)* left ventricular end-systolic volume (indexed to BSA), *LVH* left ventricular hypertrophy, *LVM(I)* left ventricular mass (indexed to BSA), *LVSV(I)* left ventricular stroke volume (indexed to BSA).

**Table 6:** Characteristics and results of the male subjects of the *mixed cohort*.

*Males*	Healthy volunteers		Fabry		LVH	
Number, *n*	64		58		103	
Age, years	32	(26–49)	42	(34–54)*	59	(46–69)*
Height, cm	181	(177–184)	178	(172–185)*	180	(174–184)
Weight, kg	80	(73–87)	77	(65–83)*	87	(78–100)*
BSA, m^2^	2.0	(1.9–2.1)	2.0	(1.8–2.1)*	2.1	(1.9–2.2)*
BMI, kg/m^2^	24	(23–27)	23	(20–26)	27	(24–31)*
LVEDV, ml	194	(172–215)	150	(133–175)*	239	(191–299)*
LVEDVI, ml/m^2^	96	(90–107)	80	(68–90)*	114	(91–151)*
LVESV, ml	77	(71–89)	39	(31–55)*	135	(87–191)*
LVESVI, ml/m^2^	39	(35–46)	21	(16–28)*	63	(41–97)*
LVSV, ml	116	(104–132)	108	(90–130)	99	(77–124)*
LVSVI, ml/m^2^	58	(53–64)	54	(48–66)	48	(37–60)*
LVEF, %	60	(56–62)	72	(67–78)*	44	(30–58)*
LVM, g	126	(113–146)	182	(148–246)*	221	(195–243)*
LVMI, g/m^2^	63	(58–70)	93	(72–127)*	103	(96–115)*
GT, mm	7.1	(6.8–7.6)	10.8	(8.7–14.3)*	10.1	(9.1–11.3)*
GTI, mm/m^2^	3.6	(3.3–3.8)	5.5	(4.5–7.1)*	4.9	(4.3–5.5)*

Only the groups that were changed from the derivation/validation cohort are shown. Characteristics for the healthy volunteers and the patient groups shown as median and interquartile range, and * denotes *p* < 0.05 compared to healthy volunteers.

*BMI* body mass index, *BSA* body surface area, *GT(I)* global wall thickness (indexed to BSA), *LVEDV(I)* left ventricular end-diastolic volume (indexed to BSA), *LVEF* left ventricular ejection fraction, *LVESV(I)* left ventricular end-systolic volume (indexed to BSA), *LVH* left ventricular hypertrophy, *LVM(I)* left ventricular mass (indexed to BSA), *LVSV(I)* left ventricular stroke volume (indexed to BSA).

When GT was corrected for body size (GTI), mean values were 3.4 ± 0.4 mm/m^2^ for females and 3.6 ± 0.4 mm/m^2^ for males, corresponding to normal ranges of 2.7–4.1 mm/m^2^ for females and 2.9–4.3 mm/m^2^ for males.

The athletes and all patient groups except for CRT candidates had higher GTI than healthy volunteers for both sexes (*p* < 0.02 for all, except for CRT candidates). LVMI for the healthy volunteers was 50 ± 7 g/m^2^ for females and 64 ± 9 g/m^2^ for males and LVEDVI was 87 ± 11 ml/m^2^ for females and 98 ± 14 ml/m^2^ for males.

The mean LVM among healthy volunteers was 2.4 SD higher in males compared to females, whereas mean GT among healthy volunteers was 2.0 SD higher for males compared to females.

### Classification of left ventricular hypertrophy subtypes

**Figure 2:**
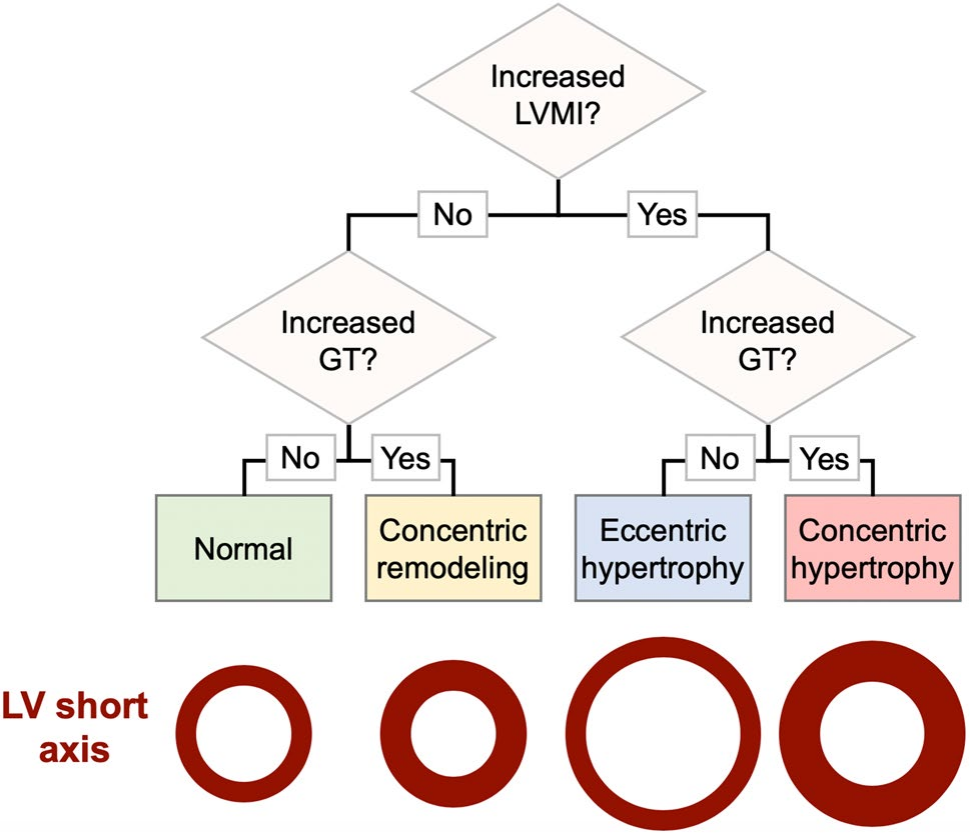
Proposed flow chart for characterizing different types of LV hypertrophy and remodeling. The bottom of the image includes a schematic illustration of a typical LV short axis slice for each classification outcome. *BSA* body surface area, *GT* global wall thickness, *LV* left ventricular, *LVMI* LV mass indexed to BSA.

As illustrated in the flow chart in Fig. 2, the combination of GT and LVMI can be used to characterize patients as being normal (normal GT, normal LVMI), or having concentric remodeling (high GT, normal LVMI), eccentric hypertrophy (high LVMI, normal GT), or concentric hypertrophy (high LVMI, high GT).

**Figure 3:**
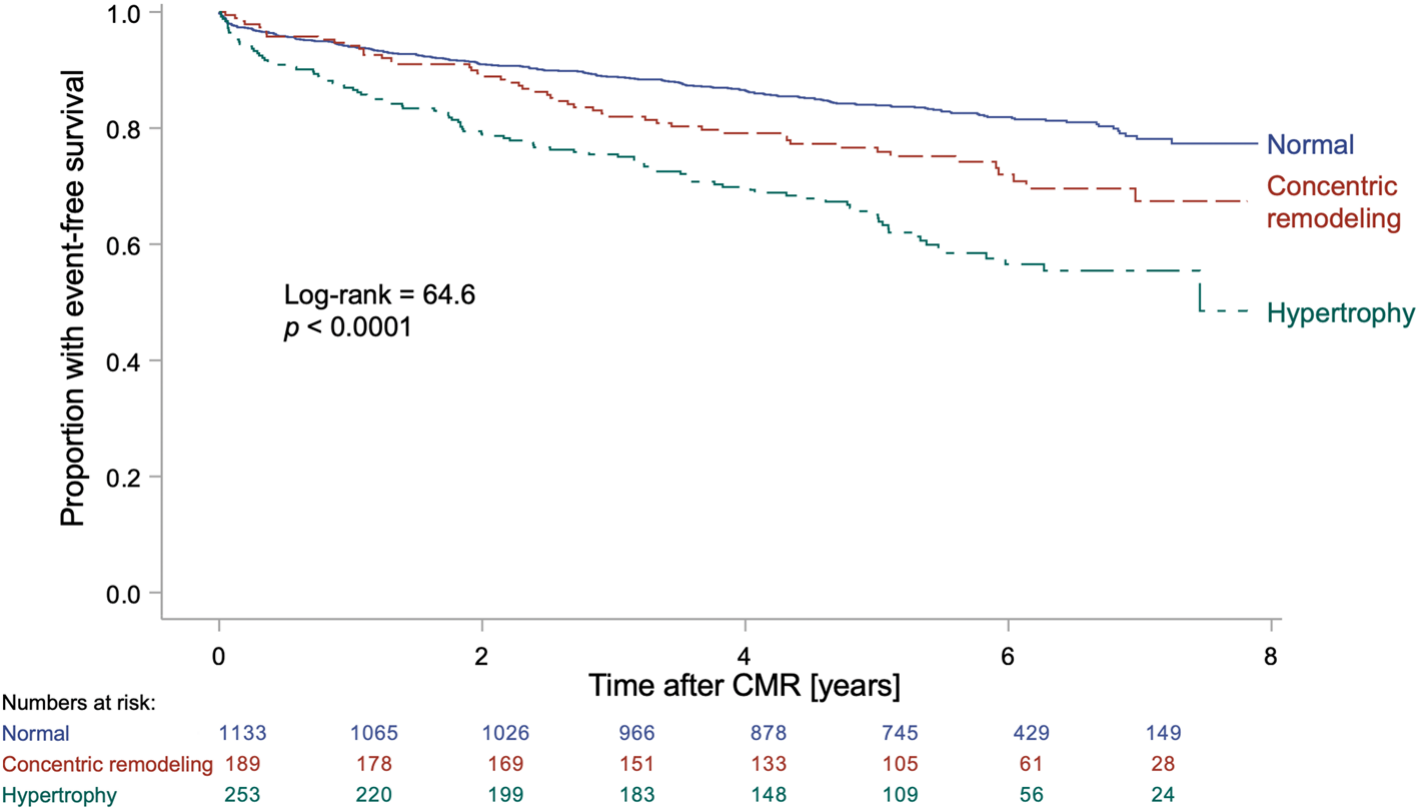
Kaplan–Meier curves for the survival cohort. Kaplan–Meier curves for the consecutive clinical patients of the *survival cohort* (*n* = 1575, follow-up 5.4 [3.9–6.4] years) classified as having either hypertrophy (increased LVMI regardless of GT as a combined group); concentric remodeling (normal LVMI, increased GT); or being classified as normal (normal LVMI, normal GT). Event-free survival was defined as absence of the combined endpoint of death or hospitalization for heart failure. The patients with hypertrophy had worse prognosis compared to both the concentric remodeling (*p* = 0.003) and the normal group (*p* < 0.0001). Patients with concentric remodeling had worse prognosis compared to the normal group (*p* = 0.004). An increase in LVMI or GT was based on the 95% upper limit of normal calculated from the healthy volunteers for females and males respectively. *CMR* cardiovascular magnetic resonance, *GT* global wall thickness, *LVMI* left ventricular mass indexed to body surface area.

In the survival cohort (*n* = 1575), the combination of GT and LVMI classified 1133 patients (72%) as being normal, 189 patients (12%) as having concentric remodeling, 89 patients (6%) as having eccentric hypertrophy, and 164 patients (10%) as having concentric hypertrophy. Patients with concentric remodeling had worse prognosis, for death or HHF, than patients classified as normal (*p* = 0.004). Both the group of patients with concentric hypertrophy and the group with eccentric hypertrophy had worse prognosis than the normal group (*p* < 0.0001 for both), and since concentric and eccentric hypertrophy did not differ from one another regarding prognosis (*p* = 0.66), they were also analyzed as one hypertrophy group (*n* = 253, 16% of all patients). These patients with hypertrophy had worse prognosis than both patients classified as normal (*p* < 0.0001) and patients with concentric remodeling (*p* = 0.003), see Fig. 3. The 5-year event rate was 16% for the patients classified as normal, 23% for the patients with concentric remodeling, 37% for the patients with eccentric hypertrophy, and 34% for the patients with concentric hypertrophy. Patient characteristics for the survival cohort are shown in Tables 7, 8, 9 and 10.

**Table 7:** Nominal characteristics for the patients of the *survival cohort*.

	Normal		Concentric remodeling		Eccentric hypertrophy		Concentric hypertrophy		*p*
*n*	1133		189		89		164		
Males	651	57%	112	59%	58	65%	90	55%	0.43
Death or HHF	202	18%	51	27%	36	40%	62	38%	< 0.001 *
Death	151	13%	41	22%	27	30%	38	23%	< 0.001 *
LGE by CMR	381	34%	90	48%	63	71%	107	65%	< 0.001 *
Infarction by CMR	216	19%	42	22%	33	37%	43	26%	< 0.001 *
Non-ischemic scar by CMR	185	16%	55	29%	34	38%	68	41%	< 0.001 *
Diabetes mellitus type 2	183	16%	70	37%	15	17%	54	33%	< 0.001 *
Hypertension	501	44%	137	72%	38	43%	117	71%	< 0.001 *
CABG prior to CMR	77	7%	23	12%	8	9%	14	9%	0.07
PCI prior to CMR	138	12%	31	16%	14	16%	19	12%	0.31

The patients are characterized as being either normal (normal GT and LVMI), or having concentric remodeling (high GT, normal LVMI), eccentric hypertrophy (high LVMI, normal GT), or concentric hypertrophy (high LVMI and GT). Data shown as *n* and percentage and *p*-value calculated using the Fisher’s exact test for difference between the groups, and * denotes *p* < 0.05. *CABG* coronary artery bypass grafting, *CMR* cardiovascular magnetic resonance, *GT* global wall thickness, *HHF* hospitalization for heart failure, *LGE* late gadolinium enhancement, *LVMI* left ventricular mass index, *PCI* percutaneous coronary intervention.

**Table 8:** Numerical characteristics for the patients of the *survival cohort*.

	Normal		Concentric remodeling		Eccentric hypertrophy		Concentric hypertrophy		*p*
*n*	1133		189		89		164		
Age at CMR, years	56	(44–65)	60	(52–68)	57	(46–65)	57	(47–66)	0.001 *
BMI, kg/m^2^	28	(24–33)	35	(29–41)	26	(23–30)	30	(25–36)	< 0.001 *
BSA, m^2^	2.0	(1.8–2.2)	2.2	(2.0–2.5)	2.0	(1.7–2.1)	2.1	(1.9–2.3)	< 0.001 *
Height, cm	173	(164–180)	173	(163–183)	173	(165–178)	173	(165–180)	0.71
Weight, kg	84	(72–98)	103	(85–122)	80	(64–93)	90	(77–109)	< 0.001 *
LVEDVI, ml/m^2^	80	(67–97)	68	(56–83)	161	(137–196)	112	(92–131)	< 0.001 *
LVESVI, ml/m^2^	32	(25–46)	26	(19–38)	123	(94–159)	62	(40–90)	< 0.001 *
LVEF, %	59	(50–64)	61	(51–66)	23	(17–32)	40	(31–56)	< 0.001 *
LVMI, g/m^2^	50	(42–59)	62	(54–72)	87	(82–93)	91	(81–106)	< 0.001 *
GT, mm	6.5	(5.7–7.2)	8.7	(7.7–9.2)	7.1	(6.6–7.8)	9.3	(8.5–10.2)	< 0.001 *
GTI, mm/m^2^	3.2	(2.9–3.5)	3.9	(3.6–4.3)	3.8	(3.4–4.1)	4.5	(4.0–4.9)	< 0.001 *
eGFR, ml/min/1.73 m^2^	90	(73–100)	85	(65–99)	81	(66–91)	81	(60–95)	< 0.001 *

The patients are characterized as being either normal (normal GT and LVMI), or having concentric remodeling (high GT, normal LVMI), eccentric hypertrophy (high LVMI, normal GT), or concentric hypertrophy (high LVMI and GT). Data shown as median (interquartile range) and *p*-value calculated using the Kruskall–Wallis test for difference between the four groups, * denotes *p* < 0.05.

*BMI* body mass index, *BSA* body surface area, *CMR* cardiovascular magnetic resonance, *eGFR* estimated glomerular filtration rate, using the MDRD formula, *GT(I)* global wall thickness (indexed to BSA), *LVEDVI* left ventricular end-diastolic volume indexed (to BSA), *LVEF* left ventricular ejection fraction, *LVESVI* left ventricular end-systolic volume indexed (to BSA), *LVMI* left ventricular mass indexed (to BSA).

**Table 9:** Nominal characteristics for the patients of the survival cohort with *normal findings*.

	All		Normal GT		High GT		*p*
*n*	326		300		26		
Female sex	147	(45%)	140	(47%)	7	(27%)	0.06
Age	47.5	(33–59)	47	(32–59)	56	(40–62)	0.04 *
Death or HHF	29	(9%)	21	(7%)	8	(31%)	< 0.001 *
Death	21	(6%)	15	(5%)	6	(23%)	0.003 *
LGE by CMR	0		0		0		-
Infarction by CMR	0		0		0		-
Non-ischemic scar by CMR	0		0		0		-
Diabetes mellitus type 2	27	(8%)	22	(7%)	5	(19%)	0.05
Hypertension	106	(33%)	91	(30%)	15	(58%)	0.008 *
CABG prior to CMR	6	(2%)	4	(1%)	2	(1%)	0.08
PCI prior to CMR	16	(5%)	11	(4%)	5	(19%)	0.005 *

Data is shown for the subgroup of the survival cohort with normal findings (no LGE, and findings within the normal range per sex for LVEDVI, LVMI, and LVEF). Age shown as median (interquartile range) and *p*-value calculated using the Kruskall–Wallis test for difference between the groups, all other data shown as *n* and percentage and *p*-value calculated using the Fisher’s exact test for difference between the groups, and * denotes *p* < 0.05. *CABG* coronary artery bypass grafting, *CMR* cardiovascular magnetic resonance, *GT* global wall thickness, *HHF* hospitalization for heart failure, *LGE* late gadolinium enhancement, *PCI* percutaneous coronary intervention.

**Table 10:** Numerical characteristics for the patients of the survival cohort with *normal findings*.

	Females		Males		*p*
*n*	147		179		
Age at CMR, years	46	(34–59)	48	(30–59)	0.9
BMI, kg/m^2^	27	(23–33)	28	(25–32)	0.05
BSA, m^2^	1.8	(1.7–2.0)	2.1	(2.0–2.3)	< 0.001 *
Height, cm	165	(160–170)	180	(175–185)	< 0.001 *
Weight, kg	72	(64–88)	91	(82–103)	< 0.001 *
LVEDVI, ml/m^2^	78	(70–86)	88	(80–98)	< 0.001 *
LVESVI, ml/m^2^	30	(27–34)	35	(31–41)	< 0.001 *
LVEF, %	61	(58–65)	60	(56–63)	< 0.001 *
LVMI, g/m^2^	45	(42–50)	58	(53–65)	< 0.001 *
GT, mm	5.8	(5.4–6.4)	7.2	(6.5–7.7)	< 0.001 *
GTI, mm/m^2^	3.2	(2.9–3.5)	3.3	(3.0–3.7)	0.002 *
eGFR, ml/min/1.73 m^2^	90	(80–105)	90	(80–105)	0.92

Data is shown for the subgroup of the survival cohort with normal findings (no LGE, and findings within the normal range per sex for LVEDVI, LVMI, and LVEF). Data shown as median (interquartile range) and *p*-value calculated using the Kruskall–Wallis test for difference between the groups, * denotes *p* < 0.05.

*BMI* body mass index, *BSA* body surface area, *CMR* cardiovascular magnetic resonance, *eGFR* estimated glomerular filtration rate, using the MDRD formula, *GT(I)* global wall thickness (indexed to BSA), *LVEDVI* left ventricular end-diastolic volume indexed (to BSA), *LVEF* left ventricular ejection fraction, *LVESVI* left ventricular end-systolic volume indexed (to BSA), *LVMI* left ventricular mass indexed (to BSA).

**Figure 4:**
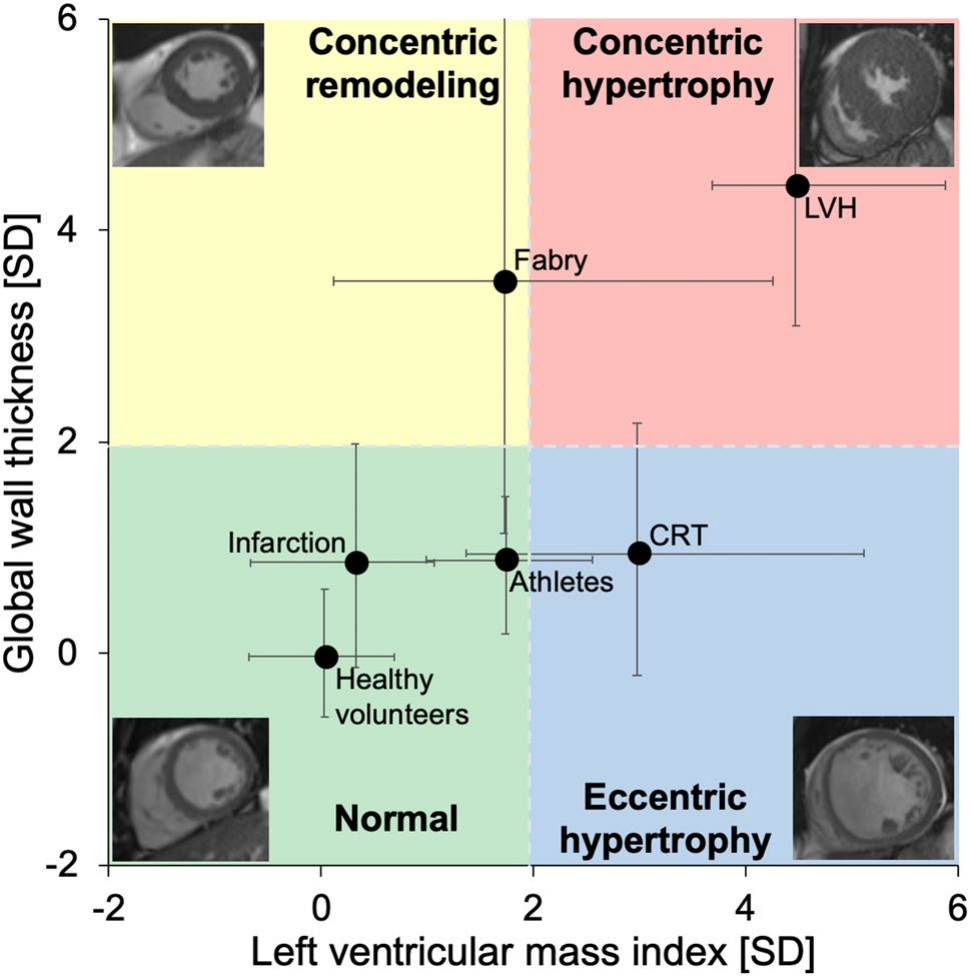
Characterization of left ventricular hypertrophy using wall thickness and mass. Global wall thickness (GT) plotted versus left ventricular mass index (LVMI) for the *mixed cohort*, who were not used in the prognostic analysis. The solid circles show the median and the whiskers show the interquartile range. Both GT and LVMI have been standardized to standard deviations (SD) from the sex-specific mean of the healthy volunteers. The colored fields show the proposed classification of hypertrophy based on LVMI and GT. The gray dashed lines indicate the upper limit of normal (+ 1.96 SD) for both GT and LVMI. The mixed cohort consists of healthy volunteers, endurance athletes, cardiac resynchronization therapy (CRT) candidates, patients with recent acute ST-elevation myocardial infarction (Infarction), patients with Fabry disease (Fabry), and patients with at least moderate left ventricular hypertrophy (LVH). Four examples of the proposed classification of hypertrophy are shown in the four corners. *CRT* cardiac resynchronization therapy, *GT* global wall thickness, *LVH* left ventricular hypertrophy, *LVMI* left ventricular mass index, *SD* standard deviations.

When evaluating the classification of the patient groups in the mixed cohort, the majority of patients with Fabry disease were classified as having concentric remodeling, albeit with a large variability due to the variability in the manifestation of the disease. The majority of patients selected for having prominent LVH were classified as having concentric hypertrophy, most CRT patients were classified as having eccentric hypertrophy, and the medians for all other groups were in the normal range, see Fig. 4.

## Discussion

This study shows that global wall thickness (GT) can be calculated from LV mass and end-diastolic volume. Similar measures have been presented previously^7^ but they need a dedicated plugin or software whereas GT can be calculated using a simple formula based on LV mass and end-diastolic volume. The prognostic analysis shows that LVMI was the most prognostic measure of hypertrophy regarding death or hospitalization for heart failure in the survival cohort as a whole, followed by GTI. GT was the most prognostic measure among patients with normal findings (normal volume, mass and ejection fraction and no scar). In this group GT was more prognostic than both age and hypertension, thus illustrating the particularly prognostic utility of GT in these otherwise normal-appearing patients. The hazard ratios for GT and GTI were larger than 1 in the univariable analysis for all examined groups indicating that a thicker wall is always a negative prognostic factor.

These findings illustrate the prognostic difference between an increased LVMI seen in advanced hypertrophy, and an increase in GT which may precede overt LV hypertrophy. Among the patients in the *survival cohort* with normal findings, GT had a higher prognostic association than both the other hypertrophy measures, as well as hypertension and age. GT can therefore be used to identify concentric remodeling in patients with otherwise normal findings and monitor these changes over time. The findings indicate that GT has a high sensitivity for detecting small changes in left ventricular wall thickness and that these small changes carry prognostic information, although the mechanism on a cellular level for this remains unclear. Furthermore, it is possible to generate an accurate and precise measure of GT from whole heart CMR measurement of LV mass and volume. The repeatability was better for GT compared to other measures, and GT was found to be less sex-dependent than LVM.

Thus, using GT together with LVMI enables characterization of different types of LV hypertrophy: normal configuration, concentric remodeling, eccentric hypertrophy, or concentric hypertrophy. This classification requires only two measures (GT and LVMI), and the results can be visualized in a two-dimensional diagram, see Fig. 4. There was no difference in outcomes between patients with eccentric and concentric hypertrophy in the survival cohort, and this is in line with the finding that LVMI was the predominant risk factor in the whole survival cohort. The distinction between eccentric and concentric hypertrophy could nevertheless imply different etiologies, necessitating different treatments.

Hypertrophy has previously been characterized using LV mass and relative wall thickness measured using echocardiography^4^ and a previous study found that the development of an abnormal LV mass and/or abnormal relative wall thickness was associated with an increased risk for cardiovascular disease^8^. Since echocardiography only measures LV wall thickness in one or two locations, this method is inherently more prone to errors compared to the proposed method. However, since GT reflects the whole LV, it will not detect areas with asymmetric hypertrophy, but such areas should be easy to identify in CMR images. Dedicated software could also be used to measure the wall thickness per slice and/or segment, but such results could not be obtained using a simple formula such as for GT.

Our study illustrates how mass and volume are geometrically related and determine wall thickness, and how both are associated with events. A previous study found that LVMI and LVEDVI were both associated with heart failure events^9^. While this is in agreement with our results, the current study clarifies how mass and volume together determine wall thickness and how both mass and wall thickness are different components of hypertrophy. Interestingly, mass and wall thickness have been shown to have distinctly different manifestations in the ECG^10^.

LV mass measured by CMR has been used to calculate measures such as mass:volume ratio^11^ and concentricity^0.67^,^12,13^ where the latter classification method requires three measures (concentricity^0.67^, LV mass, and LVEDV) for characterizing hypertrophy. By comparison, the proposed classification method is more simple since it only requires LVMI and GT (calculated from LVM and LVEDV). Concentricity^0.67^ performed well in the prognostic analysis, and the way that it is calculated is justified since an object that is twice as large in every direction will have a volume that is eight (2^3^) times larger, whereas the surrounding shell will only be four (2^2^) times as large. Indeed, calculating concentricity^0.67^ is somewhat similar to calculating GT (LVM vs LVM^0.84^, and LVEDV^−0.67^ vs LVEDV^−0.49^). Notably, GT has an advantage over concentricity^0.67^ since GT provides the average wall thickness in mm, which is an intuitive measure.

GT was increased above the upper limit of normal in 14% of the patients with normal mass in the survival cohort. Previous studies using echocardiography and relative wall thickness to define concentric remodeling have found the prevalence of concentric remodeling to be 19%^4^ and 16%^14^ among hypertensive patients with normal LV mass, which is effectively similar to our results for GT.

### Limitations

The expression used to calculate GT uses both LVM and LVEDV derived from cine images. Separate data for papillary muscles and trabeculations was not available in the present study. These structures were not included in LVM and were included in the blood volume. Therefore, the derived equation for GT is only applicable to results from exams where delineations are performed in a similar fashion. As with all cohort studies, the *prognostic cohort* is subject to some selection bias, but nevertheless reflects clinical practice and therefore remains inherently worthy of study. Only measures related to hypertrophy, as well as age, and no measures of systolic function were included in the prognostic analysis since the objective was to find a measure related to hypertrophy that is complementary to LVM, and not to identify the most prognostic measure overall.

It has been found that in healthy volunteers with blood pressure ≤ 140/90 mmHg, both mass and volume decrease with increasing age^15^. However, calculations based on data from that publication show that there was negligible change in GT with age based on GT calculated from different age group means in that study [data not shown]. Thus, normal values for GT regardless of age are appropriate.

Only linear regression was used in the prognostic analysis. The finding that the hazard ratios for GT and GTI were larger than 1 for all examined groups does not, per se, indicate a relationship that is linear, but it indicates at least that a thicker wall implies a worse prognosis overall. Multiple linear regression was performed for LVMI and GT(I) where the latter is dependent on the first (correlated with an R^2^ of roughly 0.5). Nevertheless, for the patients with normal findings, the χ^2^ was much higher for GT in both the univariable and the multivariable analysis indicating a superior prognostic performance. There were only 29 events in the group of patients with normal findings corresponding to 9% of the patients included. While the survival analysis in this subgroup is from a somewhat limited sample size, the results were nonetheless clearly statistically significant with a substantial margin (*p* < 0.001).

The aim of the study was specifically to compare the relative prognostic strength of measures related to hypertrophy. Therefore, ejection fraction, scar burden and other measures were not included in the prognostic analysis.

The present study includes exams performed at several different centers, on several different platforms, and analyzed by different observers, which can contribute to variation in the results. However, CMR measurement of LVM has a measurement precision that vastly exceeds LVM measurements by 2D echocardiography^16^, and thus variability in measurement of LVM and LVEDV would not be expected to have a sizeable impact on our results in comparison to the variability of echocardiographic approaches. Also, the test–retest repeatability was better for GT compared to LVM and LVEDV.

## Conclusions

LV mass (LVMI) is the most prognostic measure for death or hospitalization for heart failure among patients investigated with CMR for cardiac disease, whereas the global wall thickness (GT, calculated simply from LV mass and end-diastolic volume), is the most prognostic measure in patients with normal CMR findings. This suggests that an optimal measurement of LV hypertrophy is a combination of LVMI (advanced disease) and GT (early disease).

## Methods

**Figure 5:**
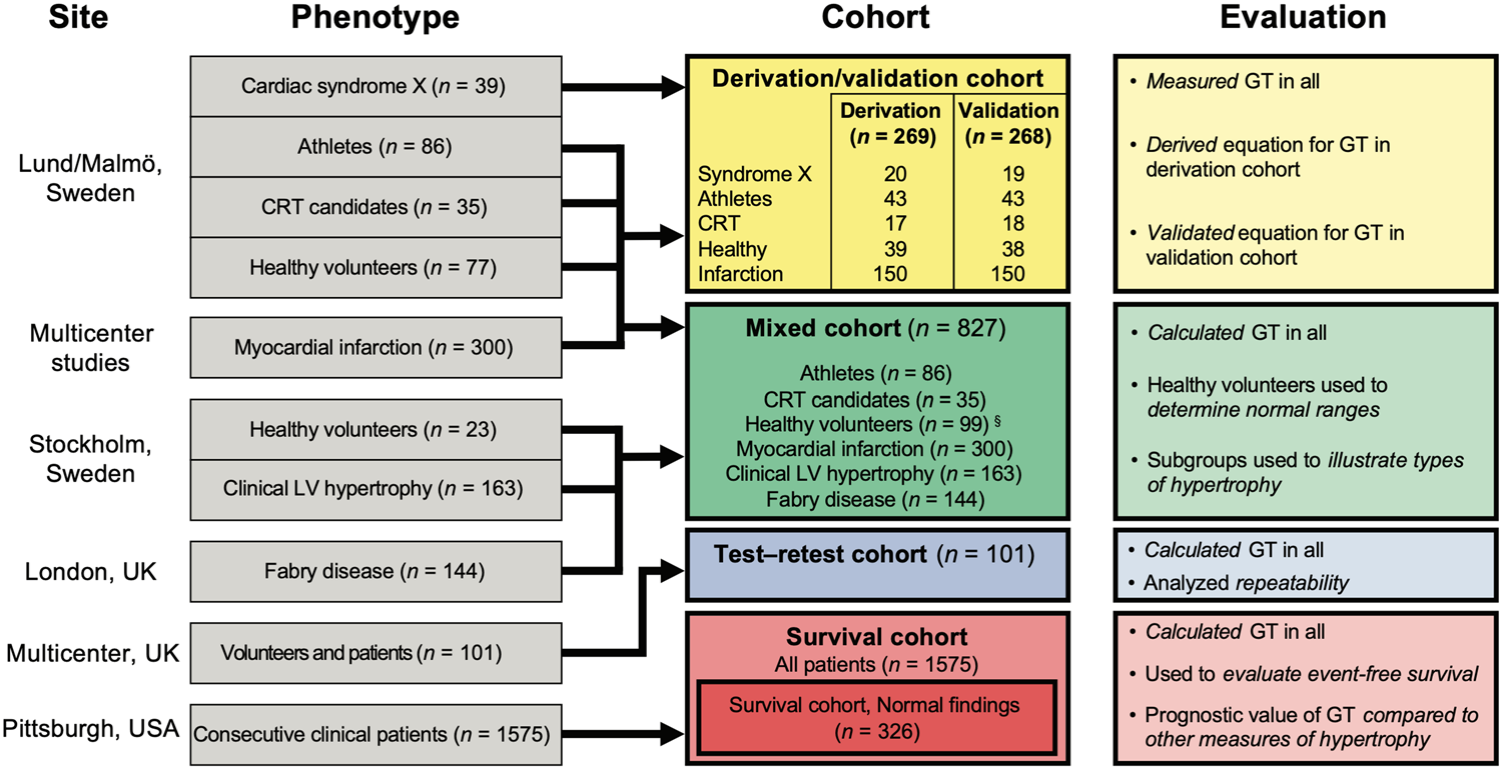
Schematic summary of the composition of the respective cohorts and what they were used to evaluate. *CRT* cardiac resynchronization therapy, *GT* global wall thickness, *LV* left ventricular. ^§^One of the healthy volunteers was an outlier and was excluded, see “Methods”.

Cohorts (total *n* = 2543) were selected from different cardiac disease states in order to address the respective aims. Figure 5 shows a schematic illustration of the composition of the cohorts and the intended use for each cohort. The data that support the findings of this study are available from the corresponding author upon reasonable request.

All research was performed in accordance with relevant guidelines and regulations. All subjects provided written informed consent and were included following approval of the local human subject research ethics review board at the respective institutions. Derivation/validation cohort: older normals (*Lundahjärta*, *Regionala Etikprövningsnämnden i Lund* (Regional Ethics board in Lund), Lund, Sweden. Dnr 741/2004 approved 22/12/2004), younger normals and athletes (*Lundahjärta med komplettering*, *Regionala Etikprövningsnämnden i Lund* (Regional Ethics board in Lund), Lund, Sweden. Dnr 269/2005 approved 16/05/2005), patients with Cardiac syndrome X (*Hjärtsvikt en diagnostisk utmaning*, *Regionala Etikprövningsnämnden* i *Lund* (Regional Ethics board in Lund), Lund, Sweden. Dnr 2013/900 approved 18/03/2014), CRT patients (*CRT Clinic* and *CRT prospektiv studie*, *Regionala Etikprövningsnämnden i Lund* (Regional Ethics board in Lund), Lund, Sweden. Dnr 2011/37 approved 17/02/2011 and Dnr 2011/550 approved 01/12/2011. ClinicalTrials.gov Identifier: NCT01426321), infarction patients (*SOCCER-studien*, *Regionala Etikprövningsnämnden i Lund* (Regional Ethics board in Lund), Lund, Sweden. Dnr 2011/258 approved 03/05/2011. Swedish Medical Products Agency EudraCT No. 2011-001452-11. ClinicalTrials.gov Identifier: NCT01423929; *MITOCARE*, *Regionale komiteer for medisinsk og helsefaglig forskningsetikk sør-øst* (Regional Committees for Medical Research Ethics South East Norway), Oslo, Norway. 2011/1423 approved 15/11/2011. EudraCT No 2010-024616-33; *CHILL-MI*, *Regionala Etikprövningsnämnden i Lund* (Regional Ethics board in Lund), Lund, Sweden. Dnr 2011/165 approved 27/04/2011. ClinicalTrials.gov Identifier: NCT01379261). Survival cohort: University of Pittsburgh Institutional Review Board, Pittsburgh, USA. MOD09010051-29 / PRO09010051 approved 24/10/2019. Test–retest cohort: NRES Committee London—Harrow, Bristol, UK. UK National Research Ethics Service 07/H0715/101 (approved 03/06/2015), 12/WM/0250, 12/YH/0551. ClinicalTrials.gov Identifier: NCT01468662. Mixed cohort: *patients* from the derivation/validation cohort above and *healthy volunteers* from the derivation/validation cohort above and from: *Regionala Etikprövningsnämnden i Stockholm* (Regional Ethics board in Stockholm), Stockholm, Sweden. Dnr 2015/2116–31/1 approved 16/02/16. Left ventricular hypertrophy (*Regionala Etikprövningsnämnden i Stockholm* (Regional Ethics board in Stockholm), Stockholm, Sweden. Dnr 2011/1077–31/3 approved 28/09/2011). Fabry disease (Northern Sydney Local Health District Human Research Ethics Committee, Sydney, Australia. HREC/16/HAWKE/125/ & SSA/16/HAWKE/299/ first approved 01/06/2016).

**Figure 6:**
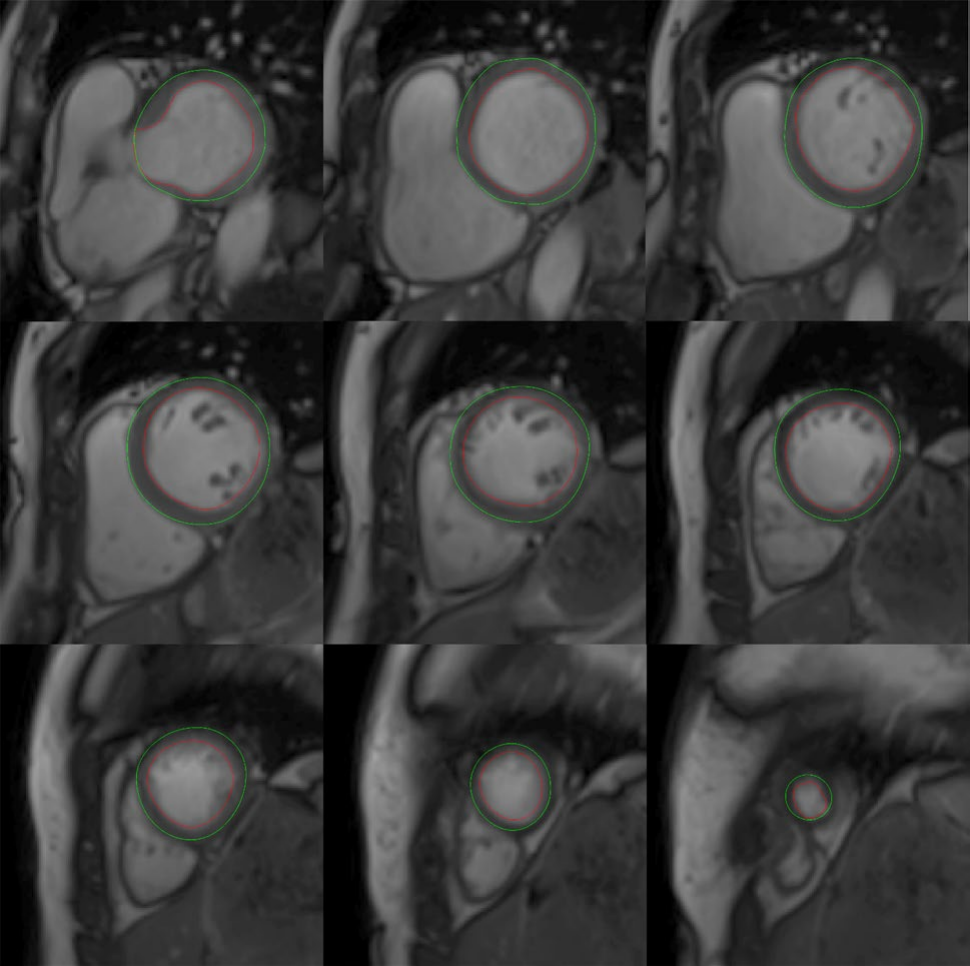
An example of a LV short axis image stack. A stack of nine short-axis cine slices (from base to apex) of the heart in end-diastole where the LV borders are outlined in green for the epicardium and red for the endocardium. The space between the epicardium and endocardium corresponds to the myocardial volume from which LV mass i calculated by multiplying the volume with the density. *LV* left ventricular.

All subjects in this study underwent cine steady-state free precession (SSFP) imaging in a LV short-axis image stack for the analysis of LVEDV and LVM, excluding papillary muscles and trabeculations, see Fig. 6 for an example of a short axis stack. The gap between the short-axis slices was between 0 and 2 mm where a difference in gap should not affect the calculations of LVEDV and LVM to any noticeable degree. BSA was calculated using the Mosteller method^17^, and GT index (GTI) as GT/BSA.

### Derivation and validation of global wall thickness

To derive and validate the new measure global wall thickness (GT), a large representative cohort of health and disease (*n* = 537) including healthy volunteers, athletes, patients with heart failure, recent infarction, cardiac syndrome X was used.

Healthy volunteers had no heart disease, no hypertension, no present or previous systemic or cardiovascular disease, were non-smokers, and did not use any medications with known cardiovascular effects, they were scanned between May 2001 and May 2009. Athletes were elite endurance athletes at national level competition in either soccer, European handball, swimming, or triathlon, they were scanned between December 2005 and March 2008. Heart failure patients were candidates for cardiac resynchronization therapy (CRT) and were scanned between December 2010 and November 2014. The patients with myocardial infarction were part of multicenter trials of acute myocardial infarction and were scanned between August 2011 and July 2015. Cardiac syndrome X patients were scanned between November 2011 and February 2016.

The athletes, the healthy volunteers and the patients with heart failure and cardiac syndrome X were all examined at Skåne University Hospital, Lund or Malmö, Sweden, and were scanned at 1.5T (Siemens Aera, Erlangen, Germany, or Philips Intera, Best, the Netherlands). For all groups, the epicardial and endocardial borders were delineated manually and the end-diastolic and end-systolic volumes as well as LVM (mean of end-systolic and end-diastolic mass) were calculated using the freely available software Segment (http://segment.heiberg.se)^18^. To evaluate systolic function in terms of ejection fraction and end-systolic volumes as well as comparing end-systolic and end-diastolic LVM was not the aim of the current study, which specifically focused on comparative prognostic measures related to LV mass and wall thickness.

**Figure 7:**
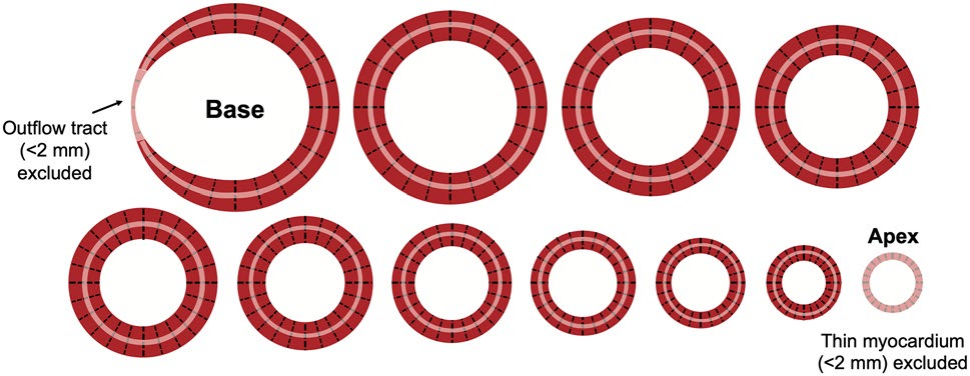
Schematic illustration of how mean left ventricular (LV) end-diastolic global wall thickness (GT) was measured using a LV short-axis image stack. The distance between the endocardial and epicardial borders at end-diastole was measured at 24 evenly distributed positions (shown as dashed black lines) around the circumference of all short-axis slices of a full LV short-axis stack from base to apex. Basal sections with a wall thickness of less than 2 mm in the LV outflow tract and the apex were excluded. The mean thicknesses for each individual short-axis slice was multiplied by the midmural circumference of that slice (pink circle), these were summed and then divided by the sum of the midmural circumference for all slices to yield the GT. *GT* global wall thickness, *LV* left ventricular.

An in-house developed plug-in for the software Segment was used to supply the measurements used to derive and validate GT. A schematic illustration of the method for measuring GT is shown in Fig. 7. The plug-in automatically measured the distance between the endocardial and epicardial borders at 24 evenly distributed positions around the circumference of each short-axis slice in end-diastole. Regions with a wall thickness of less than 2 mm in the left ventricular outflow tract or apex were excluded. The mean wall thickness of each short-axis slice was multiplied by the midmural circumference of the respective slice, and the resulting sum for all slices was divided by the sum of the circumferences of all slices to yield the GT, thereby weighting wall thickness by slice size.

In order to be able to *calculate* the GT from known parameters without the use of dedicated software, a simple equation was proposed. Since GT geometrically can only depend on cardiac mass and volume, GT was mathematically expressed as relating to LVM and LVED according to the following equation:

2 $$GT = A + B \cdot LVM^{X} \cdot LVEDV^{Y}$$

where GT is mean left ventricular end-diastolic global wall thickness in millimeters, LVM is left ventricular mass in grams, and LVEDV is left ventricular end-diastolic volume in milliliters, and *A*, *B*, *X*, and *Y* are constants to be estimated. The included groups were split into a derivation and validation cohort matched for sex and diagnosis. Matlab (R2016, Mathworks, Natick, Massachusetts, USA) was used to identify best fits for *A*, *B*, *X* and *Y* in Eq. 2. Fit performance was estimated by least squares of data against all possible combinations of *A*, *B*, *X* , and *Y* in the derivation cohort, with performance evaluated subsequently in the validation cohort. The patient characteristics of the derivation and validation cohorts are shown in Tables 1 and 2.

Once derived, both GT and other measures related to hypertrophy such as mass:volume ratio, and concentricity^0.67^ in Ref.^12^ were calculated for all subjects in order to compare their diagnostic and prognostic abilities. The remodeling index (RI) defined as (LVEDV)^1/3^/max wall thickness^19^ was calculated as (LVEDV)^1/3^/GT since maximum segmental wall thickness was not available.

#### Survival cohort

Prognostic analysis was performed in a separate cohort (*n* = 1575) of consecutive clinical patients investigated for cardiac disease. These patients underwent clinical CMR scans at 1.5 T (Siemens Magnetom Espree, Erlangen, Germany) between June 2010 and March 2016 at the University of Pittsburgh Medical Center (Pittsburgh, Pennsylvania, USA). The scan included cine SSFP imaging and late gadolinium enhancement (LGE) 10 min after a 0.2 mmol/kg dose of intravenous contrast agent (ProHance, Bracco Diagnostics, Stillwater, Minnesota, USA). This cohort has been examined regarding prognosis in several published studies^20^ but has never been studied with the objective of classifying hypertrophy. Patients with amyloidosis were excluded as they have a distinctive phenotype, and have prognostic features that markedly differ from other diseases. Exams that had previously been found to be of a sub-standard quality were also excluded.

All continuous imaging measures of LVH derived from imaging, as well as age and hypertension, were examined regarding their prognostic value. A composite end-point was used, where the time from CMR exam to hospitalization for heart failure (HHF) or death from all causes was determined as previously described, and where HHF was confirmed by two cardiologists blinded to CMR data^21^. Since both ventricular dilatation, low ejection fraction, and hypertrophy are important risk factors associated with heart failure^1,22^, the analysis was performed both for the whole cohort as well as in the subgroup of patients with completely normal findings defined as LVEDVI, LVMI, and LV ejection fraction (LVEF) within the normal ranges, and absence of myocardial scar. The normal ranges used to select this subgroup were derived from the healthy volunteers described above.

#### Test–retest cohort

In order to evaluate the repeatability of the old and new measures, a separate multicenter cohort (*n* = 101) including different pathologies as well as healthy volunteers was used. The subjects were scanned at different magnetic field strengths at several locations in the United Kingdom between September 2010 and May 2019. These subjects were all scanned on two occasions, 96% were performed within one week and 79% on the same day. All analyses were undertaken by one expert observer.

#### Mixed cohort

To obtain robust normal values, additional healthy volunteers (*n* = 23, 39% female) underwent CMR at 1.5 T (Siemens Aera, Erlangen, Germany) at Karolinska University Hospital (Solna, Sweden) between April and August 2016 and were added to the healthy volunteers from the *derivation/validation cohort,* resulting in a total of 100 healthy volunteers. One of the female volunteers had an abnormal GTI four standard deviations above the mean GTI of the other female volunteers, in spite of having a LV mass and volume within the normal range. This outlier case was therefore not used for determining normal ranges. The resulting group of healthy volunteers (*n* = 99, 35% female) was used to determine sex-specific 95% normal ranges for GT, GTI, LVEDVI, LVMI, and for the other measures listed above.

In order to illustrate the performance of the new measures for additional specific diagnoses, two additional groups of patients were included in the *mixed cohort*. Patients with prominent LVH (*n* = 163, 37% female), were selected as having LVMI more than three standard deviations above the normal sex-specific mean calculated from the healthy volunteers, and underwent CMR at 1.5 T (Siemens Aera, Erlangen, Germany) at Karolinska University Hospital (Solna, Sweden) between October 2013 and November 2015. The second group was patients with genetically confirmed Fabry disease (*n* = 144, 60% female), and were examined at 1.5 T (Siemens Avanto, Erlangen, Germany) at The Heart Hospital (London, United Kingdom) between May 2011 and July 2016. These groups were not included in any other cohort. Although the cohorts are inhomogeneous, they were included for different and well-defined purposes, as per Fig. 5.

### Statistics

Calculations were performed using IBM SPSS Statistics (version 25, IBM Corporation, Armonk, New York, USA). Data are reported as mean ± standard deviation (SD) or median [interquartile range] as appropriate. Normal ranges were defined as the range between the mean-1.96 SD and the mean+1.96 SD. To combine comparison of both sexes, measures were standardized to the sex-specific normal mean and reported as SD. The Mann–Whitney *U* test was used to compare non-normal distributions. Survival analysis was performed using SAS 9.4 (SAS Institute, Cary, North Carolina, USA). Cox regression was used, and univariable Cox regression χ^2^ values and hazard ratios (HR) were compared for different CMR parameters, standardized using the normal mean and SD per sex. The χ^2^ values were used to rank the different measures. The influence of different measures were also compared using multivariable Cox regression. Kaplan–Meier curves were plotted for the different types of hypertrophy and differences in prognosis were evaluated using the log-rank test. Test–retest variability was calculated as the relative Dahlberg error^23^ and the *F*-test was used to compare variances. Differences in prevalence were evaluated using the χ^2^-test. Statistical significance was defined as *p* < 0.05.

## Data Availability

Data can be made available upon reasonable request to the corresponding author.

## Abbreviations

CMR: Cardiovascular magnetic resonance
CRT: Cardiac resynchronization therapy
GT(I): Global wall thickness (indexed to body surface area)
HHF: Hospitalization for heart failure
LV: Left ventricular
LVEDV(I): Left ventricular end-diastolic volume (indexed to body surface area)
LVEF: Left ventricular ejection fraction
LVH: Left ventricular hypertrophy
LVM(I): Left ventricular mass (indexed to body surface area)
MVR: Mass:volume ratio
SSFP: Steady-state free precession
